# Association between feline immunodeficiency virus and *Leishmania infantum* infections in cats: a retrospective matched case-control study

**DOI:** 10.1186/s13071-022-05230-w

**Published:** 2022-05-10

**Authors:** Vito Priolo, Marisa Masucci, Giulia Donato, Laia Solano-Gallego, Pamela Martínez-Orellana, Maria Flaminia Persichetti, Ana Raya-Bermúdez, Fabrizio Vitale, Maria Grazia Pennisi

**Affiliations:** 1 Dipartimento di Scienze Veterinarie, Università di Messina, Messina, Italy; 2 Departament de Medicina i Cirurgia Animals Facultat de Veterinaria, Universitat Autònoma de Barcelona, Bellaterra, Barcelona, Spain; 3 Facultad de Medicina Veterinaria, Universidad de Córdoba, Campus Rabanales, Córdoba, Spain; 4 Centro di Referenza Nazionale per la Leishmaniosi (CReNaL), Istituto Zooprofilattico Sperimentale della Sicilia “A. Mirri”, Palermo, Italy

**Keywords:** Feline immunodeficiency virus, Leishmaniosis, Feline retrovirus, Coinfection, Risk factors, Logistic models, Polymerase chain reaction, Indirect fluorescent antibody technique

## Abstract

**Background:**

Feline leishmaniosis caused by *Leishmania infantum* is often associated with feline immunodeficiency virus (FIV) infection; however, the role and clinical significance of this coinfection remain unknown. This study aimed to assess whether FIV is associated with *L. infantum* infection in cats from canine leishmaniosis endemic areas and to report the clinical signs and hematological alterations associated with coinfection.

**Methods:**

A retrospective matched case-control study (ratio 1:2) was conducted. Data of clinical examination and complete blood count (CBC) were selected from a cohort of 705 cats examined for epidemiological studies on feline leishmaniosis conducted between 2012 and 2019. Ninety-one FIV seropositive cases and 182 FIV seronegative control cats were selected. Matching was done according to age, sex, lifestyle and geographic provenience of case cats. Rapid ELISA devices were mainly used to detect anti-FIV antibodies. Anti-*Leishmania* IgG antibodies were detected by indirect-immunofluorescence test (IFAT). *Leishmania* DNA was searched in blood, oral and conjunctival swabs by quantitative real-time PCR.

**Results:**

Feline immunodeficiency virus seropositive cats had no hematological abnormalities suggestive of an advanced stage of FIV infection and were statistically more frequently IFAT positive, and their risk of being *L. infantum* antibody positive was 2.8 greater than in the FIV seronegatives. The association of FIV seropositivity with *L. infantum* antibody positivity was confirmed in the univariable model of logistic regression. A multivariate model found FIV infection and *L. infantum* PCR positivity as predictors of a positive *L. infantum* IFAT result. Male outdoor cats from rural or suburban areas were at risk for FIV and *L. infantum* antibody positivity. Clinical signs more frequently associated with the coinfection were oral lesions, pale mucous membranes and low body condition score (BCS).

**Conclusions:**

This study documents that FIV seropositive cats with no hematological abnormalities suggestive of an advanced stage of FIV infection are more prone to be *L. infantum* seroreactive by IFAT in endemic areas. Therefore, FIV seropositive cats should be tested for *L. infantum* antibodies and treated for preventing sand fly bites. Pale mucous membranes, low BCS and oral lesions but no CBC abnormalities were significantly associated with the coinfection.

**Graphical abstract:**

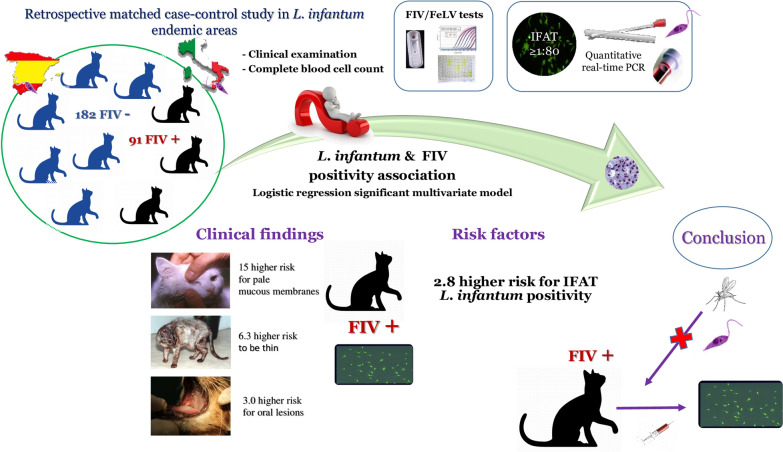

**Supplementary Information:**

The online version contains supplementary material available at 10.1186/s13071-022-05230-w.

## Background

Leishmaniosis is a vector-borne disease caused by protozoa of the *Leishmania* genus transmitted by sand fly bites. *Leishmania infantum* is the most widespread species and is of zoonotic concern, with dogs considered the main domestic reservoir in endemic areas. However, other domestic and wild animals are reported to be infectious to sand flies [1]. In recent years, an increasing number of case reports of feline leishmaniosis (FeL) and subclinical infections caused by *L. infantum* were documented in endemic areas of the Mediterranean basin [2], and FeL is considered an emerging feline disease [3]*.*

Many studies have confirmed that feline *L. infantum* infection is not negligible in areas where canine leishmaniosis is endemic [2]. However, different levels of endemicity and type of population under study or differences in diagnostic methodologies may be responsible for the high variability in antibody or molecular prevalences reported in published studies [2]. Investigations have been performed on *L. infantum* prevalence in cats in Southern Italy for a long time, reporting an antibody prevalence between 6.9 and 59% [4–10] and a molecular prevalence between 1.3 and 61% [5, 7–9, 11, 12]. In Spain, the antibody prevalence reported ranges between 3.2 and 4.8% in the Madrid area [13–16], between 2.2 and 16% in the northeast [17–19] and 28.3% in the south of the country [20]. The molecular prevalence reported ranges between 0 and 0.43% in the Madrid area [13–16], between 3 and 26% in the northeast [6, 17–19, 21] and 25.7% in the south of the country [20].

Feline immunodeficiency virus (FIV) is a retrovirus distributed in feline populations worldwide and associated with adult, male and free-roaming cats because the main transmission path is via biting [22]. Prevalence rates of FIV positivity are therefore influenced by the characteristics of populations under study, and surveys reporting FIV prevalence in the same area of South Italy investigated in the present study found a wide range of positivity, between 7.6 and 37% [6, 8, 23]. FIV prevalence studies available from various regions of Spain reported ranges between 5.1 and 20.9% [13–15, 17, 19, 24, 25].

Among risk factors for feline *L. infantum* infection, co-infection with feline immunodeficiency virus is the most investigated. Many studies found a significant association between FIV and *L. infantum* positivity in cats [5, 9, 10, 12, 19, 26–28]. However, other studies did not document this association [8, 14, 17, 20, 29–32]. Therefore, the association between FIV and *L. infantum* infection in cats remains unclear. The aims of the present study were to assess whether FIV and *L. infantum* infections are associated in cats living in canine leishmaniosis endemic areas of South Italy (Calabria and Sicily) and Spain (Catalonia and Andalusia) and to investigate clinical signs and hematological abnormalities associated with this coinfection.

## Methods

### Study design, cat characteristics and selection

A retrospective matched case–control study was carried out. Feline immunodeficiency virus seropositive (cases) and FIV seronegative (controls) cats were selected from our research database if they were evaluated by physical examination and tested for FIV as well as for *L. infantum* by IFAT and PCR in blood, conjunctival and oral swabs with the same methodology. A population of 705 cats studied between 2012 and 2019 was examined. The assumptions were: alpha < 5%, power ≥ 80%, two controls per case, *L. infantum* positivity 17.3% (prevalence of the population where cases and controls were extracted from) and expected odds ratio (OR) of 2.5. The target sample size was 92 cases and 184 controls. Cases and controls were matched according to age (> 6 months and exposed to sand flies for at least one transmission season), sex (male/female), lifestyle (indoor/outdoor) and geographical provenience (Sicily, Calabria, Catalonia, Andalusia) (Table 1). Cats were enrolled after their owners provided informed consent and in compliance with the requirements of ethics committees from the authors' academic institutions. The “Strengthening the Reporting of Observational Studies in Epidemiology” (STROBE) recommendations were followed to describe the study methods [33].

**Table 1:** Signalment and history data of 91 feline immunodeficiency virus (FIV)-positive cases and 182 FIV-negative controls, with description of number (N) and percentage (%) of cats recorded for each variable

Variable	Cases		Controls		Total	
	N	(%)	N	(%)	N	(%)
Sex						
Male	61	(67)	122	(67)	183	(67)
Female	30	(33)	60	(33)	90	(33)
Reproductive status						
Neutered	44	(48.3)	79	(43.4)	123	(45.1)
Not neutered	47	(51.7)	103	(56.6)	150	(54.9)
Hair						
Short	59	(64.8)	126	(69.3)	185	(67.8)
Medium	18	(19.8)	21	(11.5)	39	(14.3)
Long	3	(3.3)	13	(7.1)	16	(5.8)
Missing	11	(12.1)	22	(12.1)	33	(12.1)
Age						
≤ 24 months	26	(28.6)	52	(28.6)	78	(28.6)
> 24 months	65	(71.4)	130	(71.4)	195	(71.4)
Geographic area						
Italy	80	(87.9)	160	(87.9)	240	(87.9)
Calabria	36	(39.6)	70	(38.5)	106	(38.8)
Sicily	44	(48.3)	90	(49.4)	134	(49.1)
Spain	11	(12.1)	22	(12.1)	33	(12.1)
Catalonia	7	(7.7)	14	(7.7)	21	(7.7)
Andalusia	4	(4.4)	8	(4.4)	12	(4.4)
Lifestyle and household						
Indoor	19	(20.9)	41	(22.5)	60	(21.9)
Outdor	72	(79.1)	141	(77.5)	213	(78.1)
Owned	19	(20.9)	45	(24.7)	64	(23.4)
Stray	32	(35.2)	57	(31.3)	89	(32.6)
Missing	40	(43.9)	80	(44)	120	(44)
Single-cat	59	(64.8)	108	(59.3)	167	(61.2)
Multi-cat	18	(19.8)	60	(33)	78	(28.6)
Missing	14	(15.4)	14	(7.7)	28	(10.2)
Environment						
Rural and suburban	32	(35.2)	67	(36.8)	99	(36.3)
Urban	53	(58.2)	98	(53.8)	151	(55.3)
Missing	6	(6.6)	17	(9.3)	23	(8.4)

### Diagnosis of feline retroviral infections

Different diagnostic tests were used in the period of time considered for the retrospective selection of matched cases and controls (2012–2019). In particular, anti-FIV antibodies were investigated with commercial kits (SNAP Combo Plus FeLV antigen and FIV antibody test, Idexx Laboratories, Westbrook, ME, USA; Pet Check FIV antibody test kit, IDEXX Laboratories, Westbrook, ME, USA; INgezim FIV, Ingenasa, Madrid, Spain). The FeLV positivity was assessed by a rapid ELISA test detecting p27 antigenemia (SNAP Combo Plus FeLV antigen and FIV antibody test, Idexx Laboratories, Westbrook, ME, USA) or by blood real-time PCR (U3 region LTR-genesig^®^ Advanced kit, Rownhams, UK). All tests were performed according to the manufacturer’s protocol.

### Leishmania infantum

#### IFAT

Antigen slides were produced by C.Re.Na.L (Centro di Referenza Nazionale per la Leishmaniosi, Palermo, Italy) using *L. infantum* strain MHOM/IT/80/IPT1. A fluoresceinated anti-cat immunoglobulin G (IgG) antibody [working anti-feline IgG (H + L)-FITC, Fuller Laboratories, Fullerton, CA, USA] was used according to Persichetti et al. (2016), and the endpoint titer of positive samples was determined preparing serial two-fold dilutions of serum starting from 1:20. The cutoff value for positivity was set at 1:80 [34, 35]. Fluorescence microscope readings were done by a unique operator (MM).

#### PCR

The EDTA blood and swab DNA was extracted using the PureLink Genomic DNA kit (Invitrogen, Life Technologies Waltham, MA, USA). Blood from a clinically healthy non-infected cat was always used as a negative control. The quantitative real-time polymerase chain reaction (RT-PCR) was carried out in a CFX96 Real-time System (Bio-Rad Laboratories s.r.l., Hercules, CA, USA) using TaqMan Master Mix (Applied Biosystems by ThermoFisher, Waltham, MA, USA) and performed as previously described [36].

### Complete blood count

Complete blood count (CBC) was performed using a laser hematology analyzer (IDEXX ProCyte Dx Hematology Analyzer, IDEXX laboratories, Westbrook, ME, USA). Reference intervals of statistically analyzed CBC parameters are listed in Additional file 1: Table S1. Blood smears were stained by May Grünwald-Giemsa staining and examined for morphological abnormalities and platelet concentration estimate.

### Variables

Data extracted from the database and included in the statistical analysis were: (1) FIV and *L. infantum* diagnostic test results: anti-FIV antibodies (positive/negative); IFAT anti-*L. infantum* antibodies (titer ≥ 1:80/≤ 1:40); *L. infantum* PCR assay (positive/negative PCR test from blood and/or swabs); *L. infantum* IFAT and PCR tests (positive/negative at both tests); (2) potential risk factors for FIV and *L. infantum* positivity from signalment and history data set: sex (male/female), age (months), age group [two different cutoff values for age group setting were analyzed as they influence the exposure of cats to a different number of transmission seasons: 24 months (≤ 24 months/> 24 months) and 36 months (≤ 36 months/> 36 months)], hair (shorthaired, medium-longhaired, longhaired), lifestyle and origin (indoor/outdoor; single-cat/multi-cat household; stray/owned), provenience (Sicily, Calabria, Catalonia, Andalusia), environment (rural and suburban/ urban); (3) clinical abnormalities more frequently detected in the 273 cats (8–37% of them): poor body condition score (BCS) (< 3.5/5: underweight, ≥ 3.5/5 normal and overweight), cutaneous (presence/absence of at least one of the following: ulcers, papules, nodules, crusts, scales and alopecia), oral (presence/absence of at least one of the following: gingivitis, stomatitis) and ocular (presence/absence of at least one of the following: blepharitis, conjunctivitis, keratitis, uveitis and panophthalmitis) lesions, enlarged lymph nodes (presence/absence), pale mucous membranes (pale/ normal); (4) CBC abnormalities: anemia (and anemia severity: low, moderate or severe), neutrophilia, neutropenia, monocytosis, lymphocytosis, lymphopenia, eosinophilia, eosinopenia, basophilia, thrombocytopenia.

### Statistical analysis

Statistical analyses were performed using Stata 16 (StataCorp LP, College Station, TX).

Data set was evaluated for normal distribution by skewness and kurtosis test. *T*-test was used in case of normal distribution and Mann-Whitney *U* test when data were not normally distributed to make comparisons. According to FIV and *Leishmania* status, differences between groups were evaluated, and the IFAT titers between cases and controls were also compared. Odds ratios (OR) and 95% confidence intervals (CI) of *Leishmania* and FIV positivity were calculated and stratified according to the other variables studied. We used a univariate logistic regression model adjusted for all variables studied, and only the statistically significant ones were reported (*P* ≤ 0.05). Multivariable models were computed using statistically significant univariate models (*P* ≤ 0.05) considering two different categories: (1) risk factors; (2) clinical signs and CBC changes. Overall, 13 sets of multivariable logistic regression were constructed. Statistical analyses of *L. infantum* tests were carried out separately for IFAT, PCR and positivity to both tests. *P*-value was considered significant for values ≤ 0.05.

## Results

### Description and analysis of clinical characteristics of cases and controls

A total of 273 cats were selected with a 1:2 case/control ratio. The 273 sera were tested for anti-FIV antibodies, and 264 of them were also tested for FeLV and found negative. In particular, 144 sera were tested with SNAP Combo Plus FeLV antigen and FIV antibody rapid test (Idexx Laboratories, Westbrook, ME, USA). One hundred twenty cats were tested for anti-FIV antibodies by Pet Check FIV antibody test kit (IDEXX Laboratories. Westbrook, ME, USA) and by blood PCR for FeLV (U3 region LTR-genesig^®^ Advanced kit, Rownhams, UK). Nine sera were tested for anti-FIV antibodies by ELISA (INgezim FIV, Ingenasa, Madrid, Spain).

The 182 controls were matched to 91 cases, and the differences between the two groups according to the matched variables were not statistically significant (Fisher’s exact test). Signalment and history data of both case and control cats are reported in Table 1. The age of cats was the unique normally distributed variable (range 7–204 months; mean 66.1 months ± 48), and the difference of mean ages (cases: 67.2 months ± 47.4; controls: 64.9 months ± 48.1) of the two groups was not statistically significant (*t*-test). The two different cutoff values used for age group settings did not yield different results, and we reported results obtained with age group cutoff set at 24 months. No statistically significant differences were found in the other clinical characteristics of the two groups of cats examined in addition to the ones used to match cases and controls (Fisher’s exact test and chi-square test). The clinical features considered in the selected cats are listed in Table 2. The proportion of cats with enlarged lymph nodes and skin lesions was higher among FIV-seropositive cats than the FIV seronegatives [Fisher’s exact test: *P* = 0.025 (OR = 1.765; 95% CI 1.05–2.944) and *P* = 0.023 (OR = 1.978; 95% CI 1.081–3.562), respectively] (Table 2). Additionally, the ratios of neutrophilia and monocytosis were higher among cases than controls [Fisher’s exact test, respectively: *P* = 0.002 (OR = 2.513; 95% CI 1.337–4.513) and *P* < 0.001 (OR = 3.661; 95% CI 1.851–7.108), respectively] (Table 3).

**Table 2:** Clinical findings considered in the 91 feline immunodeficiency virus (FIV)-positive cases and 182 FIV-negative controls, with description of number (N) and percentage (%) of cats recorded for each variable

Variable	Cases		Controls		Total	
	N	(%)	N	(%)	N	(%)
Body condition score						
Normal or overweight	60	(65.9)	113	(62.1)	173	(79)
Underweight	20	(22)	26	(14.3)	46	(21)
Total	80	–	139	–	219	–
Missing	11	(12.1)	43	(23.6)	54	(19.7)
Mucous membranes						
Normal	80	(87.9)	166	(86.5)	246	(91.8)
Pale	9	(9.9)	13	(7.1)	22	(8.2)
Total	89	–	179	–	268	–
Missing	2	(2.2)	3	(1.6)	5	1.8
Lymph nodes^a^						
Enlarged	37	(40.7)	52	(28.6)	89	(33)
Normal	52	(57.1)	129	(70.9)	181	(67)
Total	89	–	181	–	270	–
Missing	2	(2.2)	1	(0.5)	3	(1.09)
Ocular lesions						
Presence	13	(14.3)	24	(13.1)	37	(13.6)
Absence	78	(85.7)	157	(86.3)	235	(86.4)
Total	91	–	171	–	272	–
Missing	0	–	1	(0.5)	1	(0.5)
Skin lesions^a^						
Presence	27	(29.7)	32	(17.6)	59	(21.6)
Absence	64	(70.3)	150	(82.4)	214	(78.4)
Total	91	–	182	–	273	–
Oral lesions						
Presence	40	(44)	46	(25.3)	86	(36.7)
Absence	50	(55)	98	(53.8)	148	(63.2)
Total	90	–	144	–	234	–
Missing	1	(1)	38	(20.1)	39	(14.3)

^a^Statistically significant Fisher’s exact test

**Table 3:** Statistically significant hematological changes in 91 feline immunodeficiency virus (FIV)-positive cases compared to 182 FIV-negative controls, with description of number (N) and percentage (%) of cats recorded for each variable

Variable	Cases		Controls		Total	
	N	(%)	N	(%)	N	(%)
Neutrophilia^a^						
Presence	28	(30.8)	32	(17.6)	60	(22)
Absence	47	(51.6)	135	(74.2)	182	(66.7)
Missing	16	(17.6)	15	(8.2)	31	(11.3)
Monocytosis^a^						
Presence	23	(25.3)	18	(9.9)	41	(15)
Absence	52	(57.1)	149	(81.9)	201	(73.6)
Missing	16	(17.6)	15	(8.2)	31	(11.4)

^a^Statistically significant Fisher’s exact test

### *Leishmania infantum* and FIV positivity association

*Leishmania infantum* IFAT and PCR results are displayed in Table 4. Cases were statistically more frequently IFAT positive than controls [Fisher’s exact test: *P* = 0.001 (OR = 2.765; 95% CI 1.482–5.249)], but the difference was not significant when considering the PCR test results or both tests (Fisher’s exact test). Feline immunodeficiency virus infection was associated only with an increased risk of *L. infantum* antibody positivity detected by IFAT, and the ORs of strata for selected covariates in FIV and IFAT *L. infantum*-positive cats are summarized in Table 5.

**Table 4:** Positivity to *L. infantum* tests of 91 feline immunodeficiency virus (FIV)-positive cases and 182 FIV-negative controls, with description of number (N) and percentage (%) of cats recorded for each variable

Variable	Cases		Controls	
	N	%	N	%
IFAT^a^				
Positive	26	(28.6)	23	(12.7)
Negative	65	(71.4)	159	(87.3)
PCR				
Positive	7	(7.7)	10	(5.5)
Negative	84	(92.3)	172	(94.5)
IFAT and PCR				
Positive	6	(6.6)	3	(1.7)
Negative	85	(93.4)	179	(98.3)

^a^Statistically significant Fisher’s exact test

**Table 5:** Odds ratios (OR), 95% confidence intervals (CI) and *P*-values for feline immunodeficiency virus (FIV) and IFAT *L. infantum* positivity (IFAT/FIV) according to the selected covariates

	OR	CI	*P*
IFAT\FIV	2.765	1.399–5.459	0.001
Covariables: risk factors			
Sex			
Female	1.978	0.536–7.052	0.001
Male	3.226	1.398–7.471	0.001
Lifestyle and environment			
Indoor	2.466	0.397–14.899	0.001
Outdoor	2.825	1.325–6.020	0.001
Urban area	2.580	1.026–6.488	0.001
Suburban/rural area	3.352	1.026–11.04	0.001
Covariables: clinical signs			
Oral lesions			
Absent	1.736	0.583–5.005	0.014
Present	3.039	1.056–9.026	0.014
Body condition score			
Underweight	6.273	1.193–41.415	0.019
Normal or overweight	1.606	0.674–3.755	0.019
Mucous membranes			
Normal	2.175	1.012–4.684	0.004
Pale	15	1.030–766.4	0.004

Medians of IFAT titers of positive cases and controls were not statistically different (Mann-Whitney *U*-test).

The results of univariable and multivariable analyses of significant risk factors and clinical abnormalities are listed in Table 6. In the univariable models, *L. infantum* antibody positivity was significantly associated with FIV antibody positivity, but this association was not found with PCR positivity (logistic regression: *P* = 0.002). A unique multivariable model yielding significant associations was obtained, and it was with *L. infantum* antibody positivity, anti-FIV antibody positivity and *L. infantum* PCR (logistic regression: *P* < 0.0001). Logistic regression models did not find significant associations of risk factors and clinical abnormalities considered for *L. infantum* and FIV-positive cats.

**Table 6:** Univariate and multivariate analyses of significant risk factors and clinical abnormalities according to *L. infantum* positivity

Variable	N	LR chi^2^	Coef	SE	*P*	CI	Model *P*
*L.infantum* IFAT	Univariate models						
FIV positivity	273	9.96	1.017	0.322	0.002	0.386–1.648	0.002
*L. infantum* PCR positivity	273	14.19	2.101	0.554	0.000	1.014–3.187	0.000
Age group	273	4.77	0.481	0.223	0.031	0.0436–0.919	0.029
Oral lesions	234	9.32	1.020	0.336	0.002	0.361–1.679	0.002
Anemia	241	4.72	0.780	0.352	0.027	0.0909–1.469	0.030
Monocytosis	242	4.32	0.824	0.385	0.032	0.0703–1.578	0.038
*L.infantum* IFAT	Multivariate model						
FIV positivity	273	23.01	0.993	0.334	0.003	0.338–1.646	0.000
*L. infantum* PCR positivity	273		2.064	0.560	0.000	0.046–3.181	
*L. infantum* PCR	Univariate models						
* L.infantum* IFAT positivity	273	14.19	2.101	0.554	0.000	1.014–3.187	0.000
Age group	273	6.05	0.947	0.405	0.020	0.152–1.742	0.014
BCS	219	7.92	-1.295	0.481	0.007	-2.239- (-0. 351)	0.005
Monocytosis	242	7.74	1.607	0.550	0.004	0.525–2.681	0.005
Eosinophilia	242	4.74	1.801	0.730	0.014	0.371–3.231	0.029

LR chi^2^, likelihood ratio chi-square test; Coef, logistic regression coefficient; SE, standard error; *P, P*-value; CI, 95% confidence interval; Model *P, P*-value model

## Discussion

### Clinical characteristics of cases and controls

Feline immunodeficiency virus is a RNA virus belonging to the family Retroviridae, subfamily Lentiviridae, a group of viruses known to cause life-long infections with protracted incubation periods [22]. Immunosuppression is determined in FIV-positive cats by a progressive decline in CD4+ T cells number, reduction in the CD4+/CD8+ ratio, generalized lymphoid depletion, reduced ability to respond to antigenic stimulation and dysregulation of cytokine production [22]. Immunosuppression contributes to secondary and opportunistic infections but FIV-positive cats remain clinically healthy for years, depending on the infecting isolate [22]. The FIV seropositive case cats of this study differed from controls regarding clinical abnormalities observed in the 273 studied cats. In particular, FIV seropositive cats more frequently showed enlarged lymph nodes and skin lesions. Peripheral lymphadenomegaly is reported in both early and more advanced stages of FIV infection and is directly caused by the virus as well as by secondary infections, immune-mediated and neoplastic conditions observed in FIV-positive individuals [22]. Miscellaneous skin diseases are described in FIV-positive cats, and they are mainly caused by secondary and opportunistic infections that may have a more severe and prolonged course compared to FIV negative cats [37]. Additionally, cutaneous neoplasms (particularly carcinomas) are reported with high rates in FIV-positive cats [37]. In the present study, FIV seropositive cats had hematological abnormalities related only to increased neutrophil and monocyte concentration. In agreement with the present results, neutrophilia [38] and monocytosis [38, 39] were the most common hematological abnormalities seen at the time of the first diagnosis in cats with naturally occurring FIV infection in previous studies [39, 40]. During the asymptomatic phase of FIV infection, the number of granulocyte/macrophage and erythroid progenitors is unchanged [40], and cats have a normal hematological response to concurrent diseases [38]. Conversely, in advanced stages of FIV infection cytopenia (anemia, leukopenia, neutropenia, and lymphopenia) is more common [40]. In this study, the stage of FIV infection was not assessed with immunological markers, and this is a limitation shared with all field studies that have so far investigated the association between FIV and *L. infantum* infections. Based on hematological findings, we assume that many of these cats were not in terminal stages of the disease. FIV-induced immunosuppression could have facilitated a secondary or opportunistic infection, to which an appropriate inflammatory response (neutrophilic and monocytic) was made [38]. However, it is important to highlight that neutrophilia is also compatible with stress leukogram, immune-mediated disorders, neoplasia and tissue necrosis and that monocytosis may also occur in many of these conditions [41].

### *Leishmania infantum* and FIV positivity association

We found that FIV antibody positivity was associated with IFAT antibody positivity to *L. infantum* in cats from areas endemic for both infections. In particular, FIV seropositive cats had a 2.8 times higher risk to be *L. infantum* antibody positive. Similarly to a previous study, we did not find differences in the antibody titer among the two groups [6]. Additionally, this association was confirmed by the logistic regression analysis performed in the univariable model and in the significant multivariable model constructed. Previous studies evaluating risk factors for *L. infantum* positivity, such as FIV seropositivity, were prevalence studies that analyzed cross-sectional data [5, 9, 10, 12, 19, 26–28, 30, 42]. Large cross-sectional studies are often based on routinely collected samples and history, and clinical findings data can be incomplete and do not provide information on some confounding factors [5]. We designed a specific case-control study to test the hypothesis that FIV seropositivity was associated with *L. infantum* positivity in endemic areas, and we were able to match cases and controls for confounding factors such as age, sex, lifestyle and geographic area. The study was retrospective, but data were obtained from a selected population of 705 cats homogeneous for systematic recording of clinical data and *L. infantum* and CBC assays. Unfortunately, methods used for testing retroviral infections were inhomogeneous, and this is a limitation of the study. However, all the different tests we used have high sensitivity and specificity and are widely used in relevant studies about feline pathogens [43–45].

As well as cross-sectional studies, case-control studies are not able to prove which of the associated variables has a causative role. This question is answered by longitudinal field investigations which unfortunately are not easily performed in veterinary medicine.

We did not find associations of FIV seropositivity with *L. infantum* PCR positivity. We performed PCR assays on three different tissues preferring non-invasive samples (swabs) and residual EDTA blood, and the number of PCR-positive cats was low when the sample size was calculated according to IFAT positivity. Consequently, results concerning PCR should be interpreted with caution, and a larger sample size should be assessed. However, in the multivariate model, PCR positivity was also a FIV seropositivity predictor for *L. infantum* antibody positivity. Since 1998, a significant association between FIV infection and anti-*L. infantum* antibodies was reported [10], and subsequently many other studies have confirmed this association with *L. infantum* antibody [12, 19, 26, 27], PCR [28, 42], antibody and/or PCR [5, 9] positivity. However, sample size and FIV prevalence were variable in these studies as well as diagnostic techniques used to evaluate both FIV and *L. infantum* positivity, and this is a limitation to compare results from different studies or make a meta-analysis. The role of FIV coinfection in *L. infantum*-positive cats could therefore be better investigated by evaluating markers useful to assay the cat immunocompetence. In *L. infantum*-infected dogs, susceptibility to the development of clinical disease is due to reduced cellular immune response and high antibody level [46]. Adaptive humoral and cell-mediated immune response is elicited by *L. infantum* feline infection, but no difference was found in FIV seropositive cats in a study evaluating the ex vivo blood production of *L. infantum*-specific IFN-γ [6].

The selected cohort of 273 cats included only cats > 6 months in order to examine cats exposed to sand flies for at least one transmission season, and we analyzed two different cutoff values for setting age groups to compare cats exposed to two or three transmission seasons with cats exposed to four or more sand fly seasons. In fact, age was often found to be a risk factor for cat *L. infantum* positivity in previous studies. In particular, cats > 12 [13, 47], 24 [7] or 36 months [9, 28, 48] were more frequently found infected. However, we did not find significant differences in *L. infantum* positivity among age groups of case and control cats, similarly to other studies comparing *L. infantum* positivity in cats of various age groups [26, 27] and where age analysis did not include age grouping [30]. Higher antibody titers in dogs are associated with the progression of infection to disease, but a longitudinal clinical, serological and parasitological evaluation of cats is needed to correctly analyze these data [46]. However, age can also influence host susceptibility to diseases in case of kittens or senior age [49, 50], but we did not set age groups with this aim. Iatta et al. [5] compared cats aged between 19 and 72 months (adults) with younger and older cats. Curiously, these adult cats had the highest risk for *L. infantum* positivity compared to the other age groups [5].

Akhtardanesh and others (2020) evidenced by a multivariate logistic regression analysis that *L. infantum* infection was more frequent in adult (particularly cats > 3 years old) and FIV seropositive cats [28]. Differently, when we stratified results of IFAT *L. infantum* and FIV seropositive cats according to selected covariates (Table 5), we found higher odds in males compared to females, in outdoor compared to indoor cats and in cats from rural and sub-urban areas compared to those from urban areas. Interestingly, males were always the sex category more frequently found positive in other studies detecting a significant sex difference in *L. infantum* positivity of cats [13, 26, 51]. Concerning lifestyle and housing, other studies found a significantly higher *L. infantum* positivity in cats from multi-cat compared to single-cat households [48], from rural compared to urban areas [51] and from colonies compared to catteries [47].

Concerning clinical findings (Table 5), the higher odds were for pale mucous membranes, low BCS and oral lesions. These latter findings are reported in feline leishmaniosis, and oral lesions are among the most frequent clinical signs observed in clinical cases [3]. Unfortunately, the clinicopathological data available from all 273 selected cats included only CBC, and no other clinicopathological abnormalities could be analyzed in the matched cats. This is a limitation of the study, and we are able to provide information only about CBC abnormalities that were not significantly associated with positivity to both pathogens.

## Conclusions

This case-control study documents that FIV seropositive cats with no hematological abnormalities suggestive of an advanced stage of FIV infection are more prone to be *L. infantum* seroreactive by IFAT in endemic areas. Therefore, FIV seropositive cats should be tested for *L. infantum* antibodies and treated to prevent sand fly bites. Pale mucous membranes, low BCS and oral lesions but no CBC abnormalities were significantly associated with the coinfection.

## Supplementary Information

**Additional file 1: Table S1. **Reference values of complete blood count (CBC) parameters statistically evaluated. RV: reference values.

## Data Availability

Data supporting the conclusions of this article are included in the report. Raw data are available from the corresponding author upon reasonable request.
