# Supplementary material for: Association between feline immunodeficiency virus and Leishmania infantum infections in cats: a retrospective matched case-control study
Source: Parasit Vectors. 2022 May 10;15:107. doi: 10.1186/s13071-022-05230-w (PMC9084934; doi:10.1186/s13071-022-05230-w)
Supplement: Supplementary file 1 — Additional file 1: Table S1. Reference values of complete blood count (CBC) parameters statistically evaluated. RV: reference values. [file 13071_2022_5230_MOESM1_ESM.docx]

**Additional file 1: Table S1.** Reference values of complete blood count (CBC) parameters statistically evaluated. RV:reference values

| CBC | RV |
| --- | --- |
| Hemoglobin  Neutrophils  Lymphocytes  Monocytes  Eosinophils  Basophils  Thrombocytes | 9.8-16.2 g/dL  1.48-10.29 K/µL  0.92-6.88 K/µL  0.05-0.67 K/µL  0.17-1.57 K/µL  0.01-0.26 K/µL  151-600 K/µL |
